# Autoantibodies from SLE patients induce programmed cell death in murine fibroblast cells through interaction with TNFR1 receptor

**DOI:** 10.1038/s41598-020-68088-x

**Published:** 2020-07-07

**Authors:** Tatiana N. Sharapova, Elena A. Romanova, Natalia V. Soshnikova, Alexey A. Belogurov, Yakov A. Lomakin, Lidia P. Sashchenko, Denis V. Yashin

**Affiliations:** 1 Laboratory of Molecular Immunogenetics of Cancer, Institute of Gene Biology RAS, Moscow, Russia 119334; 2 Shemyakin-Ovchinnikov Institute of Bioorganic Chemistry RAS, Moscow, Russia 117997; 3 Lomonosov Moscow State University, Moscow, Russia 119991

**Keywords:** Biochemistry, Immunology

## Abstract

Various pathological processes are known to be associated with the production of IgG autoantibodies, which have high affinity for self-antigens and often cause tissue injury and the development of autoimmune diseases. However, the mechanism of their cytotoxic activity is not clearly understood yet. Here, we have shown that the action of these autoantibodies on cells expressing TNFR1 (the cell surface receptor for TNFα) can cause both caspase-dependent apoptosis and necroptosis of these cells, with suppression of apoptosis resulting in switching to RIP1-dependent necroptosis. Analysis of necroptotic mechanisms has shown that a critical point of necroptosis is phosphorylation of RIP1 and RIP3 kinases, which is followed by the involvement of lysosomes and mitochondria in this process. The induction of cytotoxicity is initiated by the interaction of autoantibodies with TNFR1, and autoantibodies can therefore be regarded as a new functional ligand for this receptor. The innate immunity protein Tag7 (PGLYRP1) described in our recent studies is also a ligand for TNFR1 and competes with autoantibodies for binding with it. Supposedly, the cytotoxic effect of autoantibodies is one of the factors responsible for autoimmune diseases that lead to tissue injury.

## Introduction

Normal human serum contains IgM antibodies capable of reacting with self-antigens, called autoantibodies (aAb). They are characterized by moderate affinity and high avidity^1,2^, have a protective effect on the immune system, and prevent the uncontrolled development of inflammation and immune response^3–5^, thereby maintaining the healthy status of the body.

There also are high-affinity aAb of IgG isotype, which arise as a result of somatic hypermutation^1^. They bind with high specificity to self-antigens and often cause tissue injury and the development of autoimmune diseases and other pathological processes^6,7^ These autoantibodies are produced by B cells that mature in long-term contact with the antigen and reach a high concentration in the serum of patients with autoimmune diseases^8^. Different classes of aAb have been described that are specific for certain autoimmune diseases. Thus, anti-DNA and antiribosomal aAb have been identified in the serum of patients with systemic lupus erythematosus (SLE) or various lymphoproliferative disorders; anti-topoisomerase I (anti-Scl-70) aAb, in scleroderma patients; anti-Jo-1 and anti-Scl-70 aAb, in patients with polymyositis^9^.

Characteristic features common to all autoimmune disorders include the synthesis of aAb, activation of self-reactive T lymphocytes, and development of inflammation^10^. However, it has been found that anti-double-stranded DNA aAb have direct cytotoxicity in vitro and can kill target cells in the absence of complement. Certain specific properties of these aAb have been revealed, which may account for their cytotoxic effect. In particular, they have both protease and nuclease activity (hence, are often referred to as catalytic aAb)^11–13^, can enter living cells, and interact with cytoplasmic and nuclear proteins^14–16^. The heavy chain sequence in some of these aAb contains a cluster of positively charged amino acids that is similar to a nuclear localization signal directing protein import into the cell nucleus^17^. Cells attacked by anti-DNA aAb have been shown to die by caspase-dependent apoptosis^13,18,19^.

Various concepts have been proposed to explain the mechanism of tumor cell death induced by anti-DNA aAb. It is considered that, upon entering the cell, these aAb can penetrate into the nucleus and cause nucleosomal DNA fragmentation or, in an as yet unclear way, induce or suppress apoptosis^18^. The cross-reactivity of anti-DNA aAb to membrane antigens^20^ suggests that they can induce the transduction of the cytotoxic signal. However, the mechanism of their cytotoxic action still remains unclear and needs a thorough analysis.

The purpose of this study was to analyze in detail the pathways of cell death induced by IgG aAb and identify the receptor responsible for cytotoxic signal transduction.

## Results

### Autoantibodies induce apoptosis and necroptosis in tumor cells

First, we analyzed the dependence of cytotoxic activity of aAb from the serum of SLE patients on the time of incubation with target cells. We used purified IgG from the serum of three patients, and all of the results below give an average for the experiments with each individual antibody fraction. The results confirmed our previous data ^13^ that cytotoxicity does not reach a saturation plateau, but its time course shows two peaks at 3-h and 20-h time points (Suppl. Figure 1). This fact suggested that aAb may induce in target cells alternative cytotoxic processes developing at different rates.

**Figure 1:**
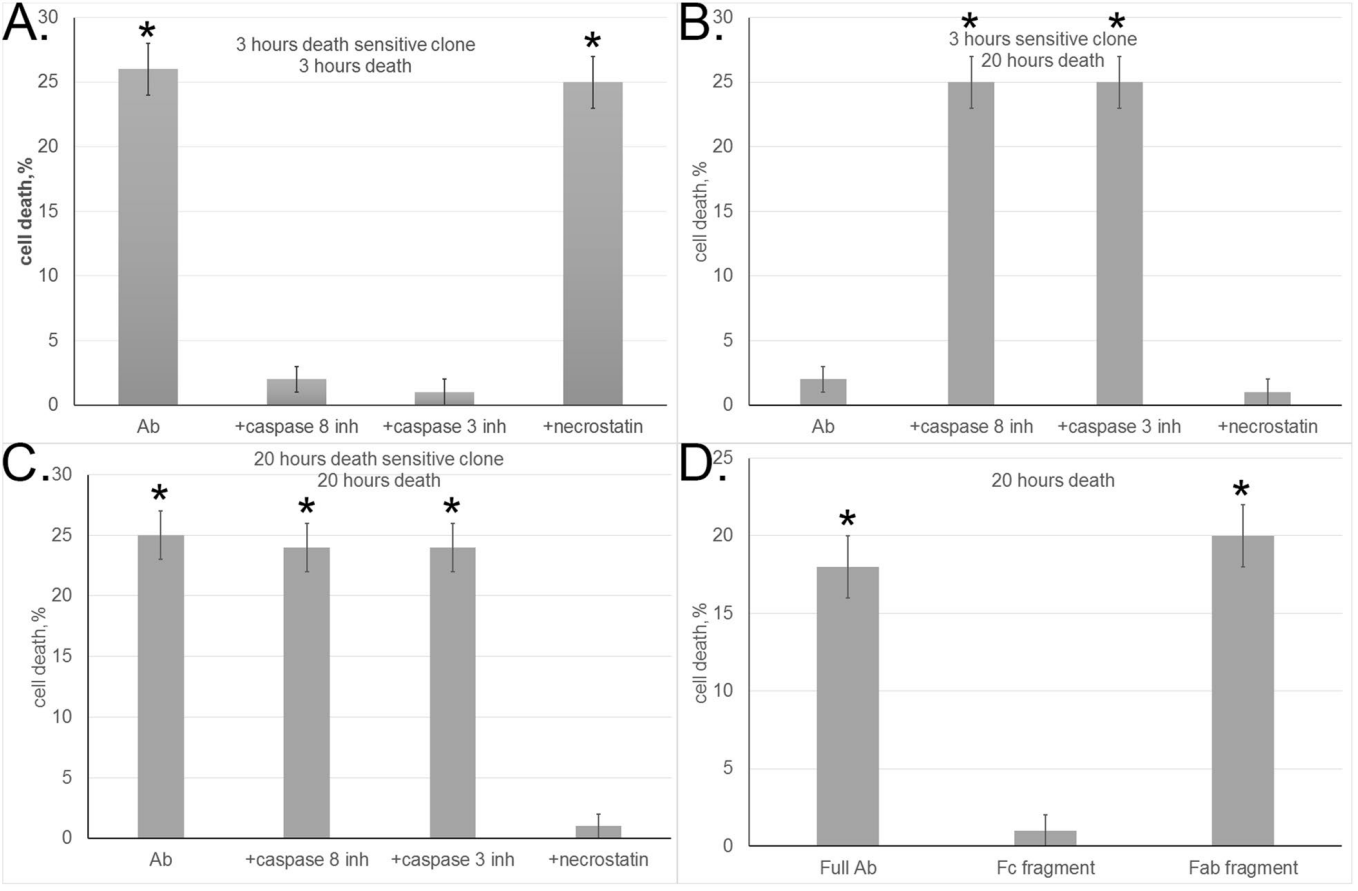
Autoantibodies induce programmed cell death. (**A**, **B**) Cell death in 3-h-sensitive L929 clone incubated with aAb alone or in the presence of caspase inhibitors and necrostatin as measured after (**A**) 3 h and (**B**) 20 h. (**C**) Cell death in 20-h-sensitive L929 clone incubated with aAb alone or in the presence of caspase inhibitors and necrostatin as measured after 20 h. (**D**) Comparative cytotoxicity of aAb and their Fc and Fab fragments (10^−8^ M) for 20-h-sensitive L929 clone as measured after 20 h.

To confirm this hypothesis, a serial dilution method was applied to the heterogeneous L929 cell population in order to isolate clones of cells that died only after either 3-h or 20-h incubation (below, referred to as 3-h-sensitive and 20-h-sensitive clones). Experiments with standard inhibitors suggested the mechanisms whereby aAb induced cell death in these clones. Cytotoxicity at the 3-h time point was blocked by specific caspase 8 and caspase 3 inhibitors (Ac-IEID-CHO and Ac-DEVD-CHO) but was insensitive to RIP1 kinase inhibitor necrostatin (Fig. 1A), suggesting that the classical caspase-dependent pathway of cell death was activated in this clone.

It should be noted that, although aAb showed no cytotoxic effect on the 3-h-sensitive clone after 20-h incubation, such an effect at this time point was observed if caspase 8 or caspase 3 activity was inhibited (Fig. 1B). Such a result suggests that the rapid and slow processes of cell death are induced by aAb through the same receptor, with suppression of caspase-dependent apoptosis leading to a changeover of the cytotoxic signal and cell death by a different mechanism.

Caspase inhibitors had no effect on cell death in the 20-h sensitive clone, but the cytotoxic activity of aAb was completely blocked in the presence of RIP1 kinase inhibitor necrostatin (Fig. 1C). It is known that phosphorylation of RIP1 kinase interacting with the cytoplasmic component of the receptor may initiate programmed cell necrosis, or necroptosis^21,22^. Therefore, it is possible that aAb may induce not only apoptosis but also necroptosis in target cells.

To find out which aAb fragment is responsible for the induction of cytotoxicity, aAb were cleaved by papain digestion into the Fab and Fc fragments. The resulting fragments were separated using Protein A Sepharose and tested for cytotoxicity. The Fc fragment proved to have no effect on target cells, whereas the cytotoxic activity of the Fab fragment was the same as that of complete aAb (Fig. 1D).

### Autoantibodies induce necroptosis with the involvement of RIP3 kinase, lysosomes, and mitochondria

To confirm the ability of aAb to initiate the necroptotic cell death pathway, we performed experiments for revealing the classical molecular mechanisms of necroptosis in aAb-treated cells.

According to published data, the initiation of necroptotic signal starts with activation of the phosphokinase cascade: autophosphorylation of RIP1 kinase causes phosphorylation of RIP3 kinase and leads to the formation of a necrosome and subsequent phosphorylation of MLKL pseudokinase (a functional RIP3 substrate), which activates a variety of necroptotic processes in the cell^23–25^.

**Figure 2:**
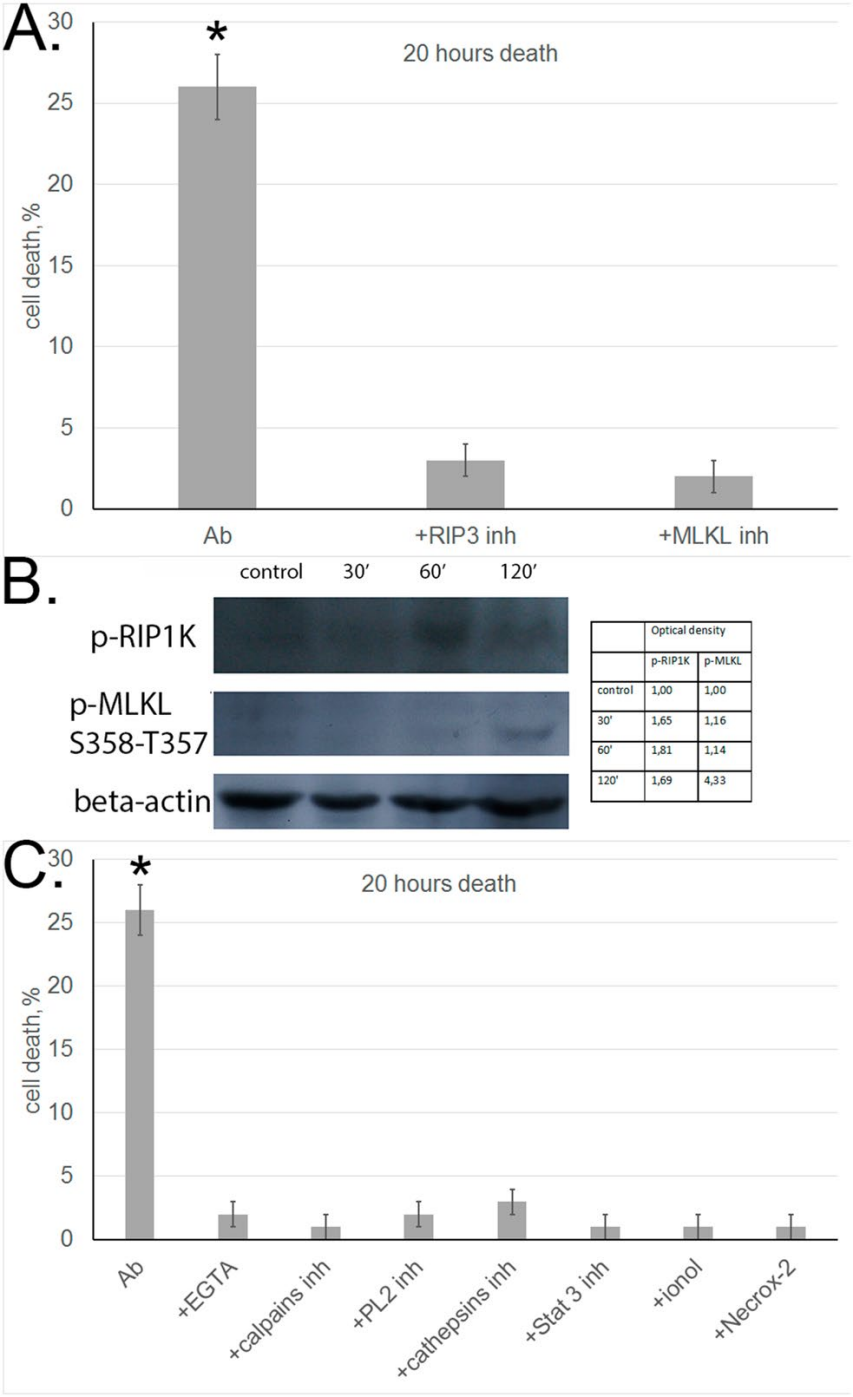
Autoantibodies induce necroptosis with the involvement of cell organelles in target cells. (**A**) Cell death of L929 cells incubated with aAb alone or in the presence of RIP1 and MLKL inhibitors for 20 h. (**B**) Western blot analysis of cytoplasmic proteins with antibodies against p-RIPK1 and p-MLKL in L929 cells incubated with aAb for different periods of time. Protein bands were quantified by using ImageJ software. The data represent the density of each band normalized to corresponding b-actin band. (**C**) Cell death of L929 cells incubated with aAb alone or in the presence of EGTA, ionol, Necrox-2 and inhibitors of calpains, cathepsins, STAT3, and PL2.

First, we used inhibitor analysis to study activation of these phosphokinases under the effect of aAb. As shown above, aAb-induced cytotoxicity dropped abruptly upon RIP1 kinase inhibition (Fig. 1C). Cell preincubation with RIP3 or MLKL inhibitors also completely abolished the cytotoxic activity of aAb (Fig. 2A). This is evidence that necrosome formation is a prerequisite for aAb-induced cell death.

To support this conclusion, experiments were performed to reveal phosphorylated forms of RIP1 and MLKL in L929 cells incubated with aAb. Western blotting with corresponding antibodies showed that phosphorylated forms of both enzymes appeared in these cells (Fig. 2B).

It is also known that phosphorylated MLKL is oligomerized into a trimer and transferred to the plasma membrane, where it promotes the opening of ion channels and stimulates the influx of Na^+^ and Ca^2+^ ions into the cell^26^. This leads to activation of Ca^2+^-dependent phosphokinases and proteases, including phosphokinase PLA2 and calpain involved ion permeabilization of lysosomal membranes. In view of these data, we analyzed the influence of reduction in Ca^2+^ concentration and inhibition of PLA2 phosphokinase and calpain activities on the development of aAb-induced necroptosis. The cytotoxic effect proved to be suppressed when the cells were incubated with aAb in the presence of specific Ca^2+^ chelator EGTA or PLA2 and calpain inhibitors (Fig. 2C).

Lysosomal membrane permeabilization leads to the leakage of lysosomal enzymes, and cathepsins released into the cytosol may retain their enzymatic activity at neutral pH (while being optimally active at acidic pH). The results shown in Fig. 2C provide evidence that cathepsin D inhibitor blocks cytotoxicity, suggesting that lysosomal enzymes and involved in programming aAb-initiated necroptosis.

Permeabilization of the mitochondrial membrane under the effect of cathepsins is regarded as one of the pathways of cytotoxic signal induction, as it leads to changes in the membrane potential and the accumulation of reactive oxygen species (ROS) on the membrane^27,28^. The role of ROS in programmed cell death was confirmed by the fact that aAb-dependent cytotoxicity was abolished when incubation was performed in the presence of antioxidants (Fig. 2C).

It is also known that the transcription factor STAT3 after phosphorylation at Ser727 is translocated to mitochondria and disturbs the functioning of the respiratory chain, which leads to ROS generation^29^. We observed no cytotoxicity when target cells were incubated with aAb in the presence of STAT3 inhibitor, which suggests that STAT3 contributes to ROS generation and accumulation (Fig. 2C).

Thus, aAb proved to initiate RIP1-dependent cell necroptosis with the involvement of lysosomes and mitochondria, as it was previously shown for necroptosis induced by the Tag7–Hsp70 cytotoxic complex^28^.

### Autoantibodies interact with TNFR1 receptor

Initiation of alternative cytotoxicity pathways under the action of the same ligand suggests that cell death is triggered through a corresponding receptor that can induce different cytotoxic processes occurring at different rates. This is characteristic of cell death receptors, particularly TNFR1 and Fas. As shown in our recent study^30^, the Tag7–Hsp70 cytotoxic complex binds to the TNFR1 receptor on L929 cells and induces their rapid death by apoptosis and relatively slow death by necroptosis.

**Figure 3:**
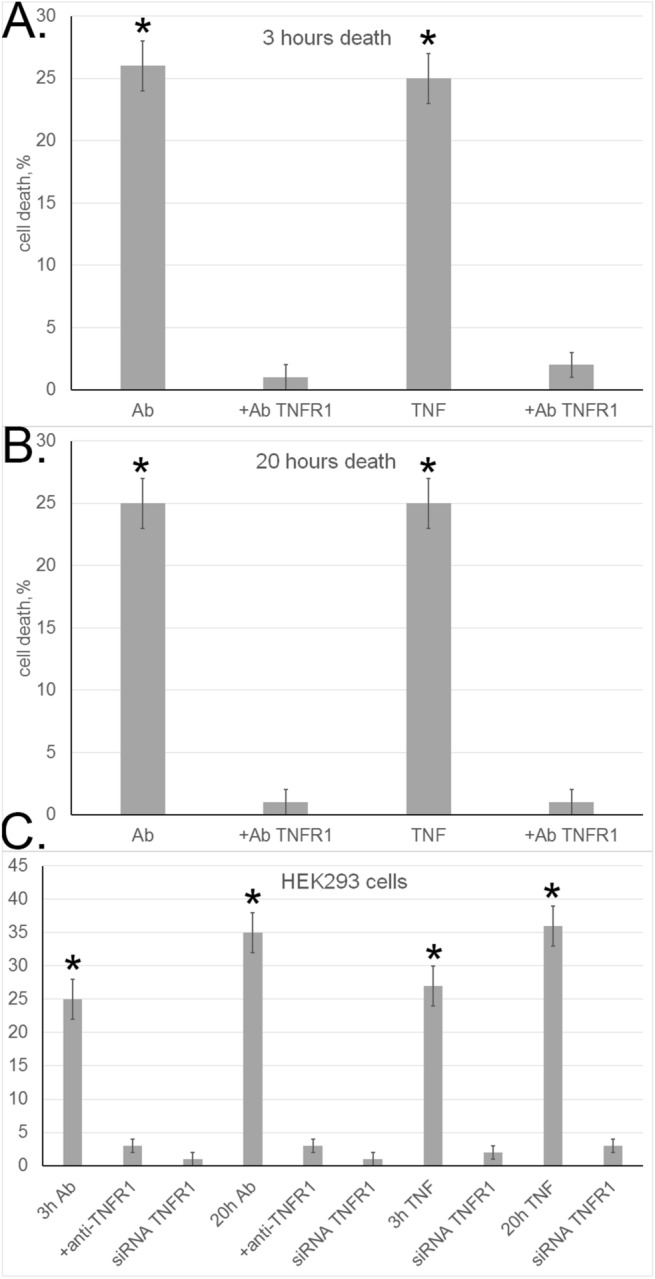
Autoantibodies induce programmed cell death through TNFR1 receptor. (**A**, **B**) Cytotoxic effects on L929 cells recorded after incubation with aAb or TNF-α alone and in the presence of anti-TNFR antibodies for (**A**) 3 h and (**B**) 20 h. (**C**) The same recorded in experiments with normal HEK 293 cells and TNFR1-knockdown HEK 293 cell line (siRNA TNFR1).

The cytotoxic activity of aAb was tested on L929 cells, which lack the Fas receptor but express the TNFR1 receptor on their surface. Hence, we supposed that apoptosis and necroptosis in these cells were induced upon aAb interaction with TNFR1. To test this hypothesis, analysis for aAb-induced cytotoxicity was performed in the presence of anti-TNFR1 antibodies, using TNFα as a cytotoxic agent in control experiments (Fig. 3A,B). As expected, the cytotoxic activity of TNFα was completely blocked by these antibodies, and the same was true of aAb-induced cytotoxicity evaluated after 3 and 20 h. To confirm these results, the cytotoxic activity of aAb was tested in similar experiments on a different cell line, HEK 293 (Fig. 3C). Cell death under the effect of aAb was also recorded at both 3-h and 20-h time points, with anti-TNFR1 antibodies blocking the corresponding cytotoxic processes.

TNFR1 gene knockdown in HEK 293 cells was performed to select clones where the level of its transcription was only 10% of that in control. These cells did not die when incubated with either TNFα or aAb; i.e., TNFR1 knockdown blocked the development of cytotoxicity. This suggests that aAb induce cytotoxicity in target cells through TNFR1.

**Figure 4:**
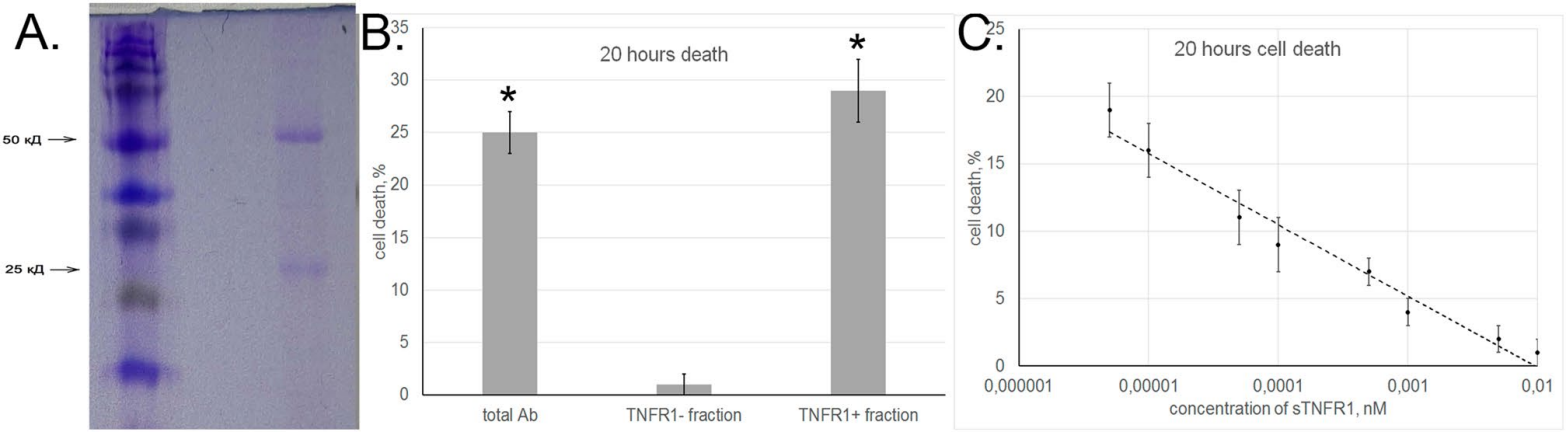
Autoantibodies interact with TNFR1. (**A**) SDS-PAGE of aAb eluted from an affinity column with sTNFR1. The bonds showed correspond to heavy and light chains of IgG. (**B**) Cytotoxicity of total aAb sample and their sTNFR1-bound and unbound fractions (TNFR1 + and TNFR1 − , respectively). (**C**) Inhibition of aAb cytotoxic activity after preincubation with different sTNFR1 concentrations.

We then tested whether aAb could interact with TNFR1. For this purpose, they were applied onto a column with the soluble extracellular fragment of TNFR1 (sTNFR1) immobilized on CNBr-Sepharose, and the bound fraction was eluted with triethanolamine. The aAb fraction specifically bound to immobilized sTNFR1 accounted for 2–5% of the total sample. Using PAGE, we have shown that the molecular weights of proteins bound to the receptor corresponded to those of IgG heavy and light chains (Fig. 4A). Tests for cytotoxic activity of bound and unbound fractions showed that this activity was characteristic only of aAb that interacted with immobilized sTNFR1 (Fig. 4B).

To confirm that aAb interacted with sTNFR1 in solution, we evaluated changes in their cytotoxic activity after preincubation with increasing concentrations of this TNFR1 fragment (Fig. 4C), as we have done earlier with TNF^30^. The results showed that the cytotoxic activity of aAb was gradually inhibited as the sTNFR1 concentration increased (IC_50_ = 2 × 10^−13^ M).

This suggests that aAb can bind with sTNFR1 in solution and that this binding could prevent their interaction with TNFR1 expressed on the cell surface.

In view of the data that cytotoxic aAb have DNA-hydrolyzing activity (12), it was relevant to find out whether TNFR1-binding aAb could cause DNA hydrolysis. To this end, we incubated plasmid DNA with aAb eluted from the column with immobilized sTNFR1 and resolved the products by agarose gel electrophoresis. The results confirmed that DNA was partially hydrolyzed (Suppl. Figure 2).

### Tag7 interferes with the interaction of autoantibodies with TNFR1

**Figure 5:**
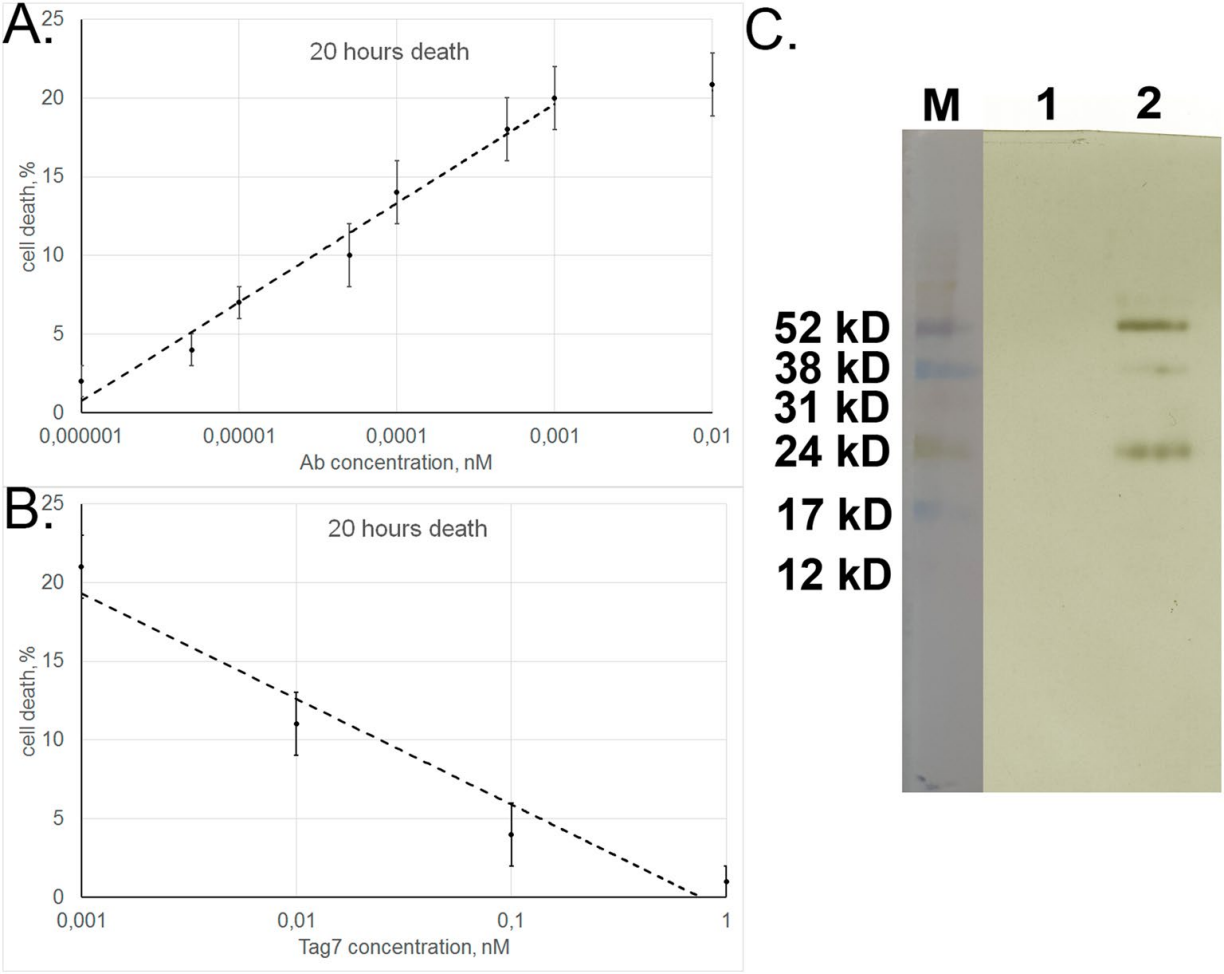
Tag7 inhibits cytotoxic activity of aAb. (**A**) Cytotoxicity of aAb depending on their concentration. (**B**) Cytotoxicity of aAb after preincubation with different concentrations of Tag7 protein. (**C**) Western blot analysis of biotinylated aAb eluted from the complex with immobilized sTNFR1: (1) washing buffer contained no aAb and (2) eluate after addition of the 100-fold excess of Tag7.

We then analyzed the dependence of cytotoxic activity of aAb on their concentration. The results show that this activity reaches a maximum at 1 × 10^−12^ M, with an IC_50_ of 5 × 10^−14^ M (Fig. 5A). This is evidence that aAb have high affinity for the extracellular TNFR1 component. The corresponding affinity of TNFα, a specific ligand for this receptor, is lower: maximum cytotoxicity is achieved at a higher TNFα concentration (5 × 10^−11^ M), with IC_50_ also being higher (2 × 10^−11^ M)^30^.

In our previous study (30), Tag7 interacting with TNFR1 was found to inhibit the cytotoxic activity of TNFα. Here, preincubation of target cells with Tag7 proved to reduce aAb-induced cytotoxicity (Fig. 5B). Therefore, it appeared likely that Tag7 interfered with the aAb–TNFR1 interaction.

To check this possibility, we performed experiments on displacing aAb from the complex with the immobilized sTNFR1 by Tag7 added in a 100-fold excess over the amount of aAb applied onto the affinity column. Antibodies applied to the column were not removed by PBS (Fig. 5C first column), but the 100-fold excess of Tag7 in PBS eluted the antibodies (Fig. 5C second column), as was shown by SDS-PAGE. Thus, Tag7 caused dissociation of aAb from the complex with TNFR1.

Analysis of the TNFR1-bound fraction by blotting with anti-Tag7 antibodies revealed the presence of Tag7 in this fraction, thereby confirming our previous data on Tag7–TNFR1 binding.

These results provide evidence that aAb have high affinity for TNFR1 and indicate that aAb and Tag7 apparently bind to the same region of this receptor.

## Discussion

The main conclusions inferred from the data presented above are as follows: (1) DNA-hydrolyzing aAb induce cell death after interacting with cell surface receptor TNFR1; (2) these aAb may be regarded as a functional ligand for this receptor, which is specific for cytokine TNFα and innate immunity protein Tag7.

Anti-DNA aAb bind to immobilized TNFR1, their cytotoxic activity is inhibited by other TNFR1-binding agents (anti-TNFR1 antibodies and Tag7) and drops abruptly upon suppression of TNFR1 transcription in the cell. All this is evidence that aAb interact with TNFR1, thereby inducing cytotoxic signal transduction.

Ligand interactions with TNFR1 can initiate alternative cytotoxic processes, committing cells to death along either apoptotic or necroptotic pathway^31^. As found previously (13, 18), cell death under the effect of DNA-binding aAb is initiated by caspase activation. The results of this study show that these aAb interact with TNFR1 and can thereby induce not only caspase-dependent apoptosis but also RIP1-dependent necroptosis in target cells.

Analysis of necroptotic mechanisms triggered by aAb has provided evidence that a critical point initiating this cell death pathway is phosphorylation of RIP1 and RIP3 kinases, which results in necrosome formation and activation of MLKL pseudokinase. Thereafter, cytotoxic signal transduction proceeds in the same way as we have shown for necroptosis induced by the Tag7–Hsp70 complex^32^: via increase in Ca^2+^ concentration, permeabilization of lysosomal membranes, release of cathepsins to the cytosol, and accumulation of ROS on mitochondrial membranes.

It is noteworthy that TNFR1-binding aAb have nuclease activity. This property and the ability of anti-DNA antibodies to enter living cells have received special attention in the recent literature. On this basis, the concept has arisen that aAb entering the cell hydrolyze DNA, with the products of its hydrolysis activating caspase-dependent apoptosis.

However, this concept is not fully applicable to our experimental system. As shown in this study, aAb can interact with the extracellular component of the TNFR1 cell death receptor and thereby induce apoptosis or necroptosis in TNFR1-expressing cells. It may well be that the mechanism of cell death induction based on the aforementioned properties of aAb is relevant only for TNFR1-negative cells.

This study considers cell death induction by aAb via TNFR1 activation in transformed cell cultures, but this receptor is also expressed on normal cells. Therefore, it appears likely that the cytotoxic effect of aAb is one of the factors responsible for autoimmune diseases that lead to tissue injury.

It is noteworthy that aAb, similar to certain cytokines, can have opposite effects in the body. On the one hand, they are responsible for autoimmune disorders that involve inflammation and tissue injury; on the other hand, they can kill tumor cells in the same way as do TNFα and the Tag7–Hsp70 complex, thereby showing an antitumor activity.

Our data show that aAb may be regarded as a functional ligand for the TNFR1 receptor specific for TNFα and Tag7 protein. They have very high affinity for the extracellular component of this receptor, with the half maximum cytotoxic concentration of aAb being lower than that of its specific ligand TNFα. This suggests that even a very low concentration of circulating aAb can have a significant effect on TNFR1-expressing cells. To gain an insight into the mechanism of TNFR1 activation by autoantibodies is essential to isolate the aAb epitope responsible for binding with TNFR1 and compare its structure with the corresponding structures of TNFα and Tag7.

## Materials and methods

### Cell lines

Experiments were performed with L929 and HEK 293 cells cultured, respectively, in DMEM and DMEM/F12 (Gibco, USA) with 2 mM L-glutamine, 10% fetal calf serum, and antibiotics (penicillin and streptomycin) at 37 °C, in an atmosphere containing 5% CO_2_. TNFR1-knockdown HEK 293 cell line and L929 clones were produced as described^30^.

### Proteins and antibodies

The study was approved by the Russian Ministry of Health and Local Ethics Committee of the V.A. Nasonova Research Institute of Rheumatology (#2018-14/17A) and was conducted in full compliance with the WMA Declaration of Helsinki, ICH GCP, and appropriate local legislation. All patients provided written informed consent at enrolment, following discussion of the study with investigators. All patients were positive in terms of anti-nuclear antibodies (ANA) test and autoantibodies toward ribonucleoprotein antigens (RNP, anti-Sm, Ro/SS-A, and La/SS-B) according to the commercial ELISAs. (See Supplemental Table 1) Level of C3 and C4 components of the complement system varied from 78 to 90 mg/dL and from 10 to 15 g/dL, respectively. No severe infection events were occurred during last 12 months in recruited patients. Sera were collected during last 2 years. Immediately after isolation each sample was frozen in liquid nitrogen and further stored at − 80 °C for no more than 2 months. Antibodies (IgGs) were purified according to (12). Briefly, IgG fraction was precipitated from the serum by 50% ammonium sulfate, followed by affinity chromatography on protein G-Sepharose (Amersham Biosciences, UK). IgG-containing fractions were then additionally purified by size-exclusion chromatography utilizing Superdex 200 (GE Healthcare, UK). The IgG amount was quantified and standardized by ELISA. IgG homogeneity and purity were verified by polyacrylamide gel electrophoresis (PAGE) stained with Coomassie and immunoblotting, under reducing and non-reducing conditions. Recombinant Tag7 was produced as described in (31). Recombinant rhTNFα was from Sigma–Aldrich (USA); polyclonal anti-TNFR1 antibodies, from Santa-Cruz (USA).

### Production of recombinant sTNFR1

Primers used to obtain the soluble component of human TNFR1 receptor were as follows:

TNFR1(sol)-for: 5′-GCATATGAGTGTGTGTCCCCAAGGAAA.

TNFR1(sol)-rev: 5′-GCTCGAGATTCTCAATCTGGGGTAGGC.

A cDNA fragment encoding amino acids 42 to 201 of TNFR1 was cloned into plasmid pET22b at *Nde*I and *Xho*I restriction sites and expressed in a bacterial system (strain BL21) induced with 1 mM IPTG 1 mM at 37 °C for 6 h. The proteins were purified on nickel nitrilotriacetic acid-agarose (Qiagen) as recommended by the manufacturer.

### Fragmentation of autoantibodies

Autoantibodies were treated with papain (Sigma–Aldrich, 10 mg/mL) at a 70:1 ratio in digestion buffer (50 mM PBS with 1 mM EDTA, pH 6.3) for 4 h at 37 °C, and then incubated with protein A Sepharose on a shaker for 1 h at 4 °C. The mixture was then centrifuged at 14,000*g* for 10 min, the supernatant containing the Fab fragment and papain was collected, and the Fc fragment adsorbed on pelleted Sepharose beads was eluted with 0.1 M glycine (pH 2.0).

### Biotinylation, affinity chromatography, immunoadsorption, and blotting

Autoantibodies were biotinylated as described^33^ and applied onto an affinity column with sTNFR1 bound to CNBr-Sepharose (Sigma–Aldrich, USA). The column was washed with PBS (pH 7.4) and 0.5 M NaCl in PBS and then eluted with 0.25 M triethylamine (pH 12). The eluted material was resolved by SDS-PAGE (31), blotted onto a nitrocellulose membrane, and the biotinylated aAb were visualized using horseradish peroxidase-conjugated streptavidin and an ECL Plus kit (GE Healthcare, USA). In experiments on aAb displacement from immobilized sTNFR1, the column was washed with PBS to which the Tag7 protein was subsequently added in a 100-fold excess over the amount of aAb in the sample. The eluted proteins were resolved by SDS-PAGE.

Phosphorylated RIP1 kinase (p-RIPK1) and MLKL pseudokinase (p-MLKL) were detected in L929 cells (3 × 10^6^ cells per sample) incubated with aAb (10^−9^ M) for 1 h. The cells were lysed and cytoplasmic proteins were isolated using RIP A buffer (Sigma–Aldrich) according to the manufacturer’s protocol. The protein preparation was resolved by denaturing PAGE^31^ and blotted onto a nitrocellulose membrane. The blots were incubated with mouse monoclonal anti-p-RIPK1 (Cell Signaling Technology, USA) or anti-p-MLKL antibodies (Abcam, UK) diluted 1:1,000, and the corresponding proteins were visualized using horseradish peroxidase-conjugated anti-mouse antibodies (1:10 000) and an ECL Plus kit (GE Healthcare). Protein bands were quantified by using ImageJ 1.52a software (https://imagej.net).

### Cytotoxicity assay

L929/HEK 293 cells were cultured in DMEM or DMEM/F12 with 2 mM L-glutamine and 10% fetal calf serum in a 96-well plate to a density of 3 × 10^4^ cells per well. The medium was then replaced by serum-free DMEM or DMEM/F12, and the cells were incubated with aAb at 37 °C, 5% CO_2_. Dead cell count was taken as described^31^ after 3 and 20 h using trypan blue staining and Cytotox 96 kit. Cytotoxicity was evaluated with regard to the death rate of control cells (not treated with aAb).

### Inhibitor analysis

Agents used to block the cytotoxic activity of aAb were as follows: caspase 3 inhibitor Ac-DEVD-CHO (5 μM), caspase 8 inhibitor Ac-IEID-CHO (5 μM), RIP1 kinase inhibitor necrostatin 1 (5 μM), RIP3 kinase inhibitor GSK 872 (5 μM), chloroquine (5 μM), NSA (5 μM), EGTA (2 μM), ionol (1 μM), Necrox-2 (1 μM), cathepsin B inhibitor Ca-074Me (10 μM), cathepsin D inhibitor Pepstatin A (10 μM), calpain inhibitor peptide (10 μM) (all from Sigma–Aldrich), STAT3 inhibitor Stattic-V (10 μM), and phospholipase C inhibitor cPLA2 (1 μM) (both from Santa-Cruz). All these agents were added 1 h prior to cell incubation with aAb.

### Statistical analysis.

An unpaired two-tailed Student’s t test was used to determine statistical significance. *P* values of less than 0.05 were considered significant (*P < 0.05; **P < 0.005). Data were analyzed using MathCad Prime 6.0 software (https://www.mathcad.com).

## Supplementary information

Supplementary information
