# Supplementary material for: Autoantibodies from SLE patients induce programmed cell death in murine fibroblast cells through interaction with TNFR1 receptor
Source: Sci Rep. 2020 Jul 7;10:11144. doi: 10.1038/s41598-020-68088-x (PMC7340778; doi:10.1038/s41598-020-68088-x)
Supplement: Supplementary file 1 — Supplementary information [file 41598_2020_68088_MOESM1_ESM.pdf]

Autoantibodies from SLE patients induce programmed cell death in murine fibroblast cells through interaction with TNFR1 receptor.

Tatiana N. Sharapova[1], Elena A. Romanova[1], Natalia V. Soshnikova[1], Alexey A. Belogurov Jr.[2,3], Yakov A. Lomakin[2,3], Lidia P. Sashchenko[1], Denis V. Yashin[1,*].

From the [1]Institute of gene biology RAS, Moscow , Russia , 119334, [2]Shemyakin-Ovchinnikov Institute of Bioorganic Chemistry RAS , Moscow , Russia , 117997 [3]Institute of Fundamental Medicine and Biology , Kazan Federal University , Kazan , Russia , 420012.

Running title: *Autoantibodies interact with TNFR1*

[*]To whom correspondence should be addressed: Denis V. Yashin, laboratory of Molecular Immunogenetics of cancer, Institute of gene biology RAS, Moscow, Russia, 119334

yashin_co@mail.ru Tel.+7(499)1359763 Fax. +7(499)1354105

Keywords: antibodies, TNFR1, apoptosis, necroptosis, Tag7, TNF, autoimmune disease, signal transduction.

Supplemental Figures and legends.

Supplemental Figure 1.

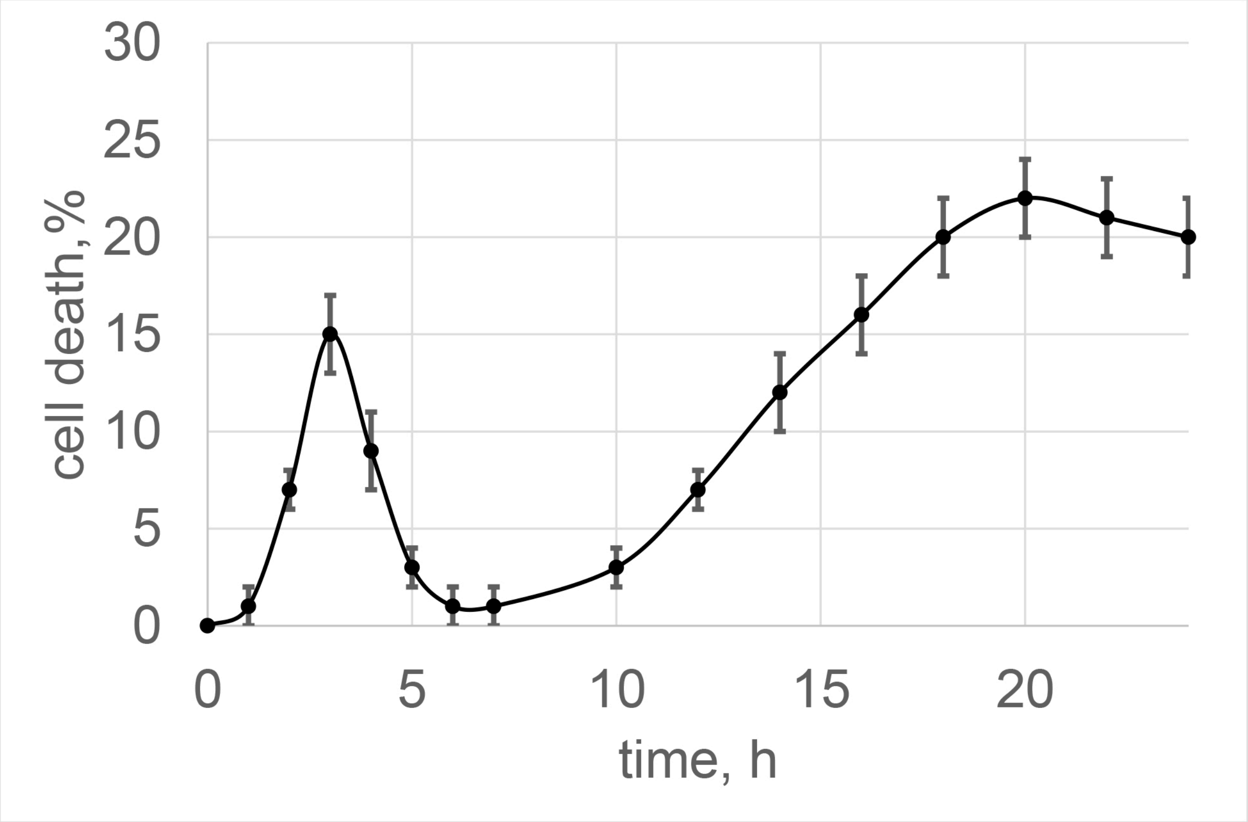

Dependency of cytotoxic activity of autoantibodies from time of incubation with target L929 cells.

Supplemental Figure 2.

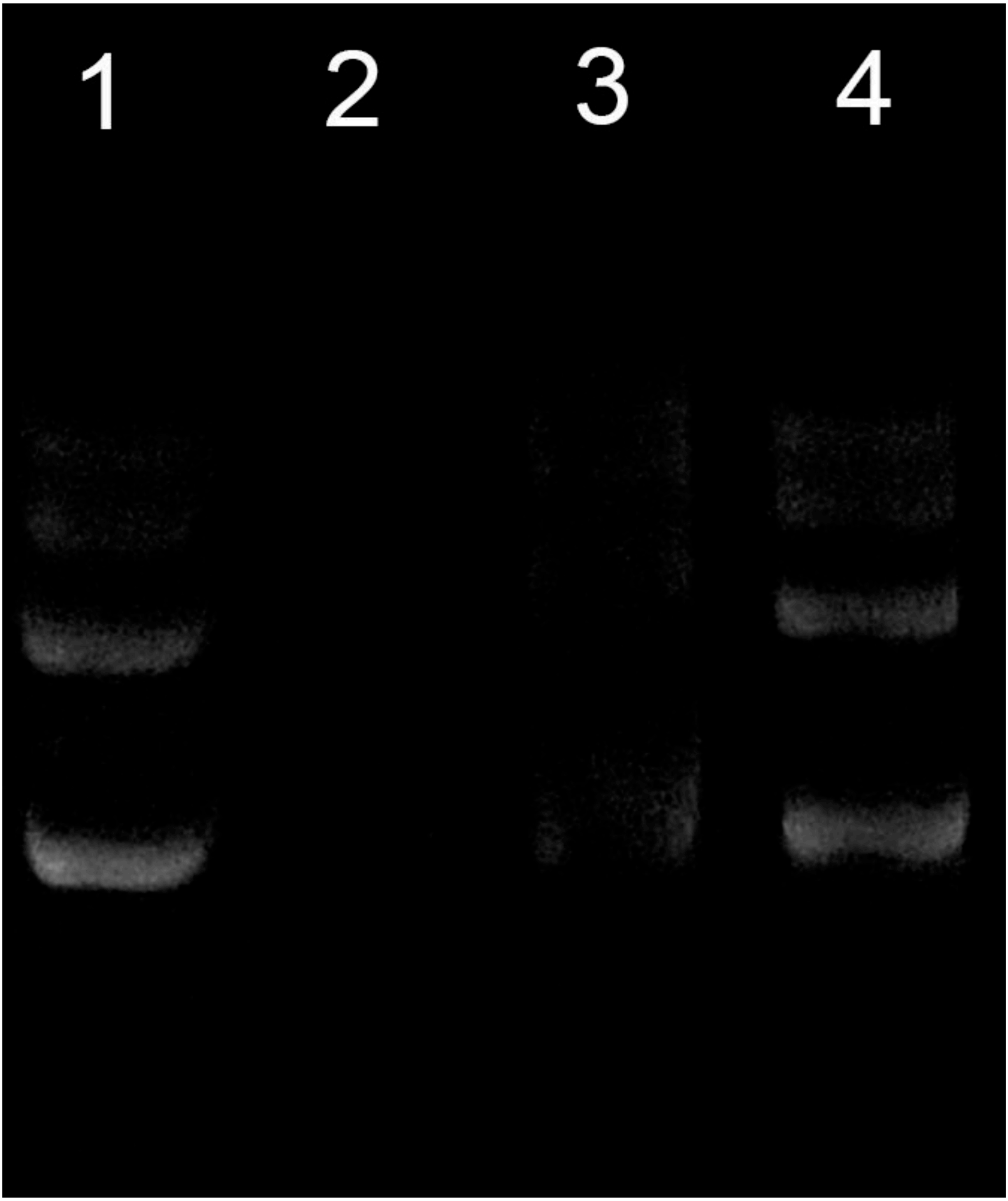

Agarose gel electrophoresis of plasmid DNA alone (1) or incubated for 1.5 h with total AA fraction (2), TNFR1 interacting fraction (3) or TNFR1 noninteracting fraction (4).

Supplemental Figure 3

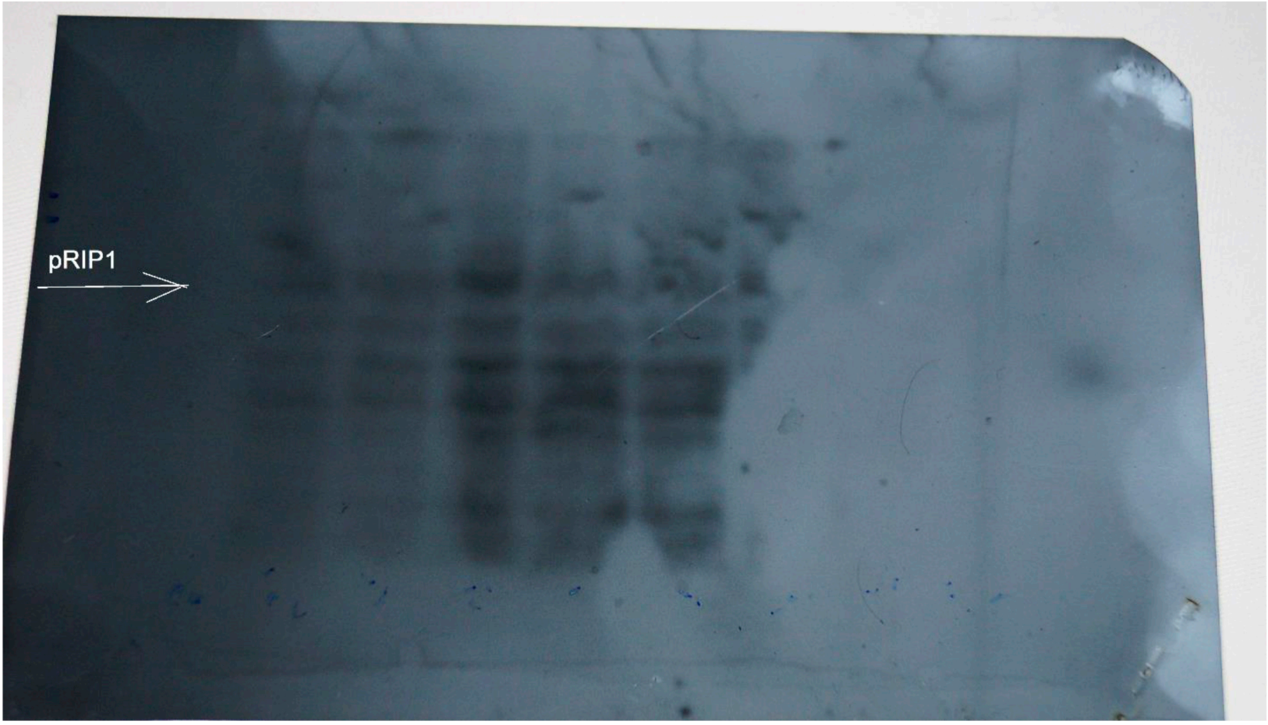

Supplemental Figure 4.

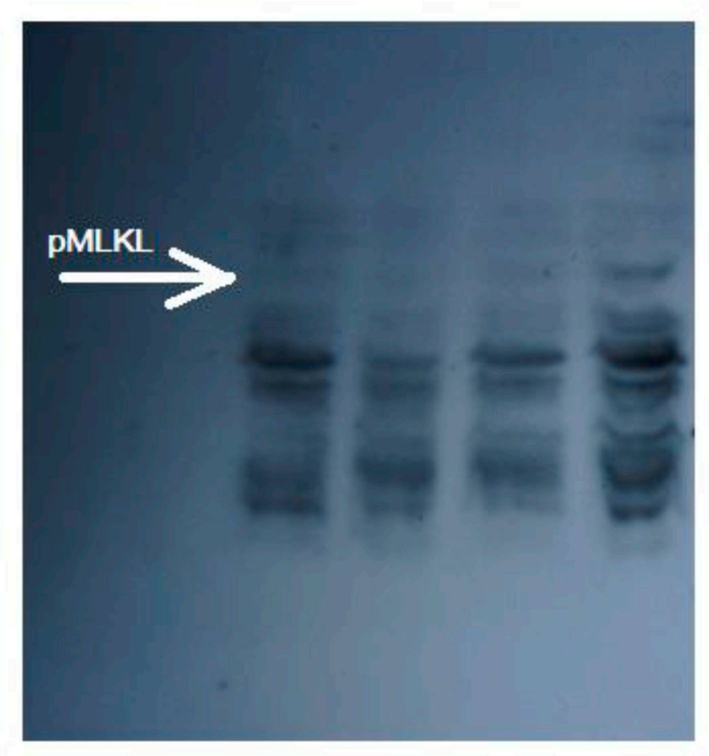

Supplemental Figure 5

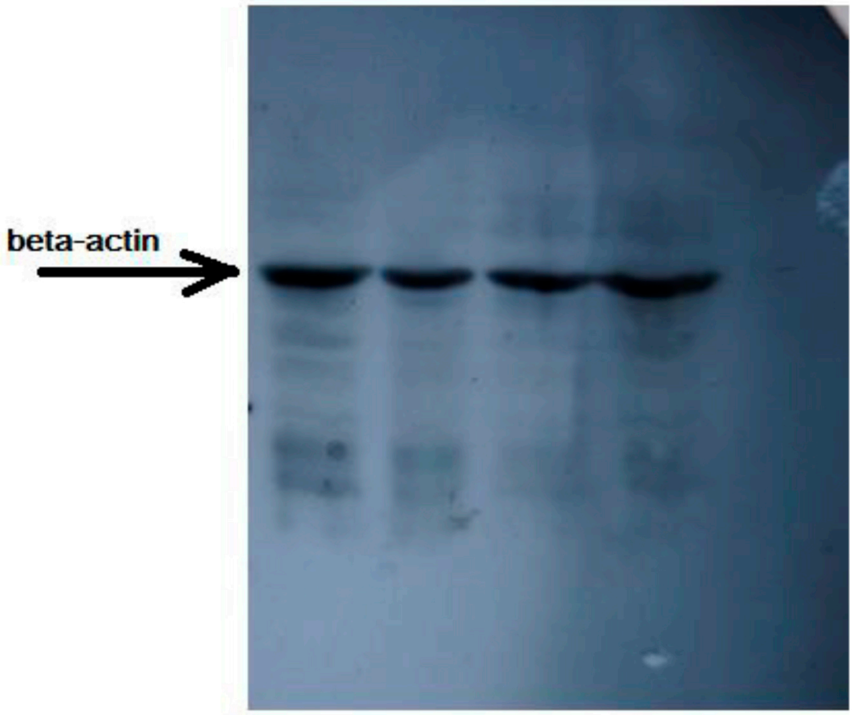

Western blot analysis of cytoplasmic proteins with antibodies against p-RIPK1 and p-MLKL in L929 cells incubated with aAb for different periods of time. Original films.

Supplemental Figure 6.

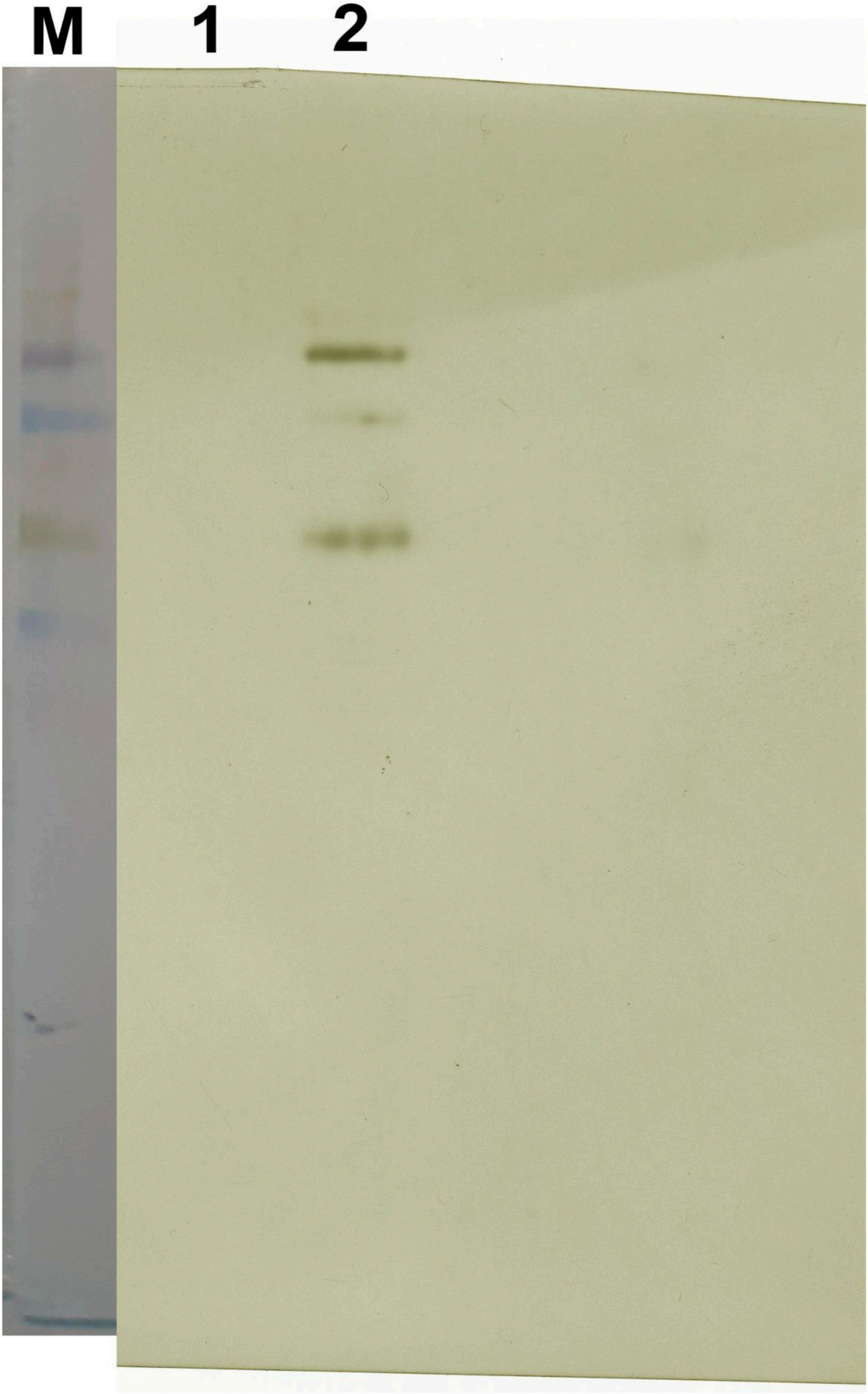

Western blot analysis of biotinylated aAb eluted from the complex with immobilized sTNFR1: (1) washing buffer contained no aAb and (2) eluate after addition of the 100-fold excess of Tag7. Original film.

Supplemental Table1.

Serum autoantibody titer of the SLE patients

Patient	ANA titer	Anti-Sm	Anti-RNP	Anti-Ro/SS-A	Anti-La/SS-B
A	1:320	1+	1+	2+	2+
B	1:320	2+	2+	2+	1+
C	1:640	1+	2+	3+	2+
